# Supplementary material for: Degradation of Sub-Micrometer Sensitive Polymer Layers of Acoustic Sensors Exposed to Chlorpyrifos Water-Solution
Source: Sensors (Basel). 2022 Feb 5;22(3):1203. doi: 10.3390/s22031203 (PMC8840410; doi:10.3390/s22031203)
Supplement: Supplementary file 1 [file sensors-22-01203-s001.zip › sensors-1546261-supplementary.pdf]

Supporting Information

Degradation of Sub-micrometer Sensitive Polymer Layers of Acoustic Sensors Exposed to Chlorpyrifos Water-Solution

David RABUS, Fanny LOTTHAMMER, Lilia ARAPAN, Joscelyn DEGRET, Frank PALMINO, Jean-Michel FRIEDT,* Frédéric CHERIOUX*

Université de Franche-Comté, FEMTO-ST, CNRS, 15B Avenue des Montboucons, F-25030 BESANCON cedex, FRANCE.

1. Deposition of PECH

PECH (825 mg) was dissolved in a solution of toluene and chloroform 50:50 (23.57g) and stirred over night at 50°C. Silicon pieces (1.5 cm x 2 cm; 380 µm) and (1.5 cm x 1.5c m; 1000 µm) were used for IR samples and AFM experiments, respectively. They were cleaned in a piranha solution for 2 min then rinsed with water. Silicon surfaces were activated by plasma (O_2, 300W, 10 min). PECH solution was then deposited on the chip surface by using a plastic pipette. The PECH solution was spread at a speed of 2500 rpm with an acceleration of 800 rpm/sec during t=30 s. They were annealed at 100°C for 2 min to obtain a clear, translucent, and homogenous polymer film with a thickness of 480 nm.

2. Deposition of PIB

2.a PIB films for AFM analysis:

PIB (825 mg) was dissolved in a solution of toluene and chloroform 50:50 (23.57g) and stirred overnight at 50°C. Silicon pieces (1.5 cm x 1.5 cm; 1000 µm) were used for AFM samples. They were cleaned in piranha solution for 2 min then rinsed with water. Silicon surfaces were activated by plasma (O_2, 300W, 10 min). PIB solution was then deposited on the chip surface by using a plastic pipette. The PIB solution was spread at a speed of 2500 rpm with an acceleration of 900 rpm/sec during t=30 s. Then, they were annealed at 100°C for 2 min to obtain a clear, translucent, and homogenous polymer film with a thickness of 690 nm.

2.b PIB films for FT-IR analysis:

PIB (236 mg) was dissolved in a solution of toluene and chloroform 50:50 (23.57g) and stirred overnight at 50°C. Silicon pieces (1.5 cm x 2cm; 380 µm) were used for IR samples. They were cleaned in piranha solution for 2 min then rinsed with water. Silicon surfaces were activated by plasma (O_2, 300W, 10 min). PIB solution was then deposited on the chip surface by using a plastic pipette. The PIB solution was spread at a speed of 2500 rpm with an acceleration of 800 rpm/sec during t=30 s. Then, they were annealed at 100°C for 2 min to obtain a clear, translucent, and homogenous polymer film with a thickness of 220 nm.

3. Acoustics detection with a PIB layer

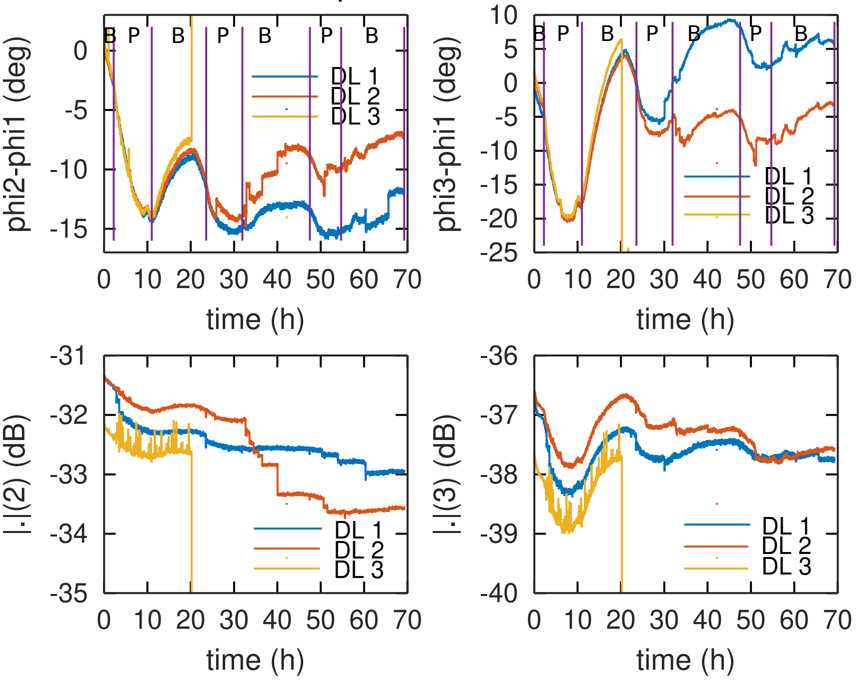

Figure S1. Evolution of the phase between echo 1 and echo 2 (top left) separated by 800 ns and echo 1 and echo 3 (top right) separated by 1600 ns. Evolution of the amplitude of echo 2 (bottom left) and echo 3 (bottom right). The measurement steps are indicated on the bottom for each chart since all steps were timestamped, with blank water-acetonitrile solution (quoted B) in which the pesticide (has been dissolved during the measurement steps named ``P''. DL: Delay line.

4. Acoustics detection with a PECH layer

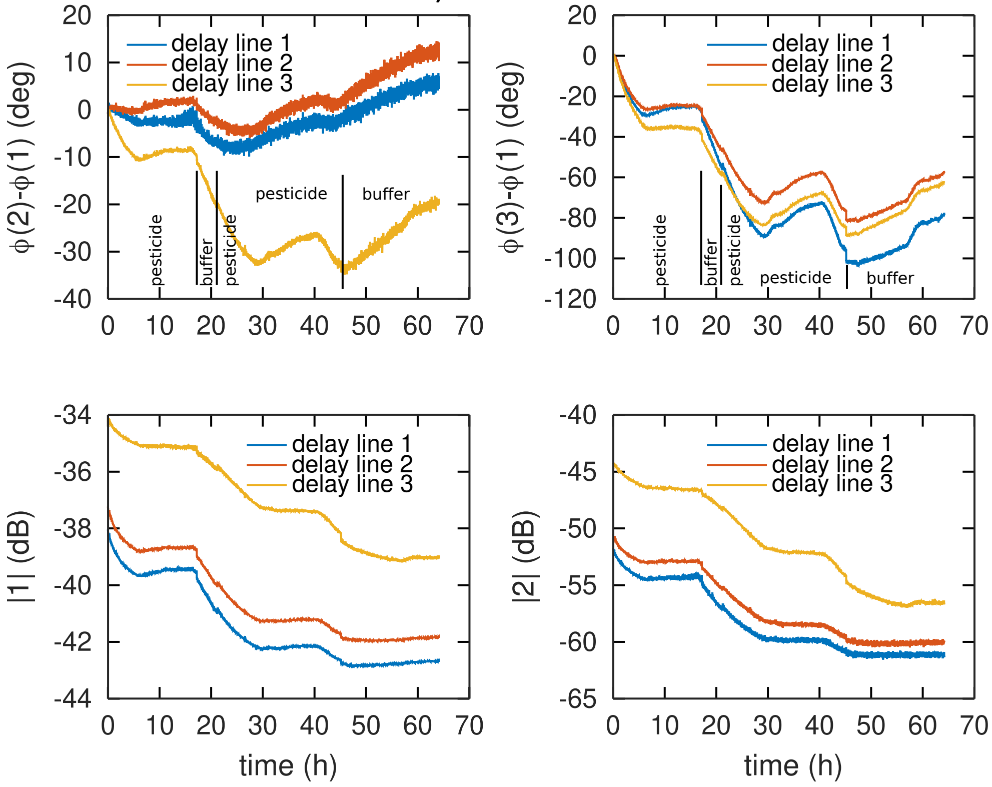

Figure S2. Evolution of the phase between echo 1 and echo 2 (top left) separated by 800 ns and echo 1 and echo 3 (top right) separated by 1600 ns. Evolution of the amplitude of echo 2 (bottom left) and echo 3 (bottom right). The measurement steps are indicated on the bottom for each chart since all steps were timestamped, with blank water-acetonitrile solution (quoted Buffer) in which the pesticide has been dissolved during the measurement steps named ``pesticide''.

5. AFM images of PIB layer

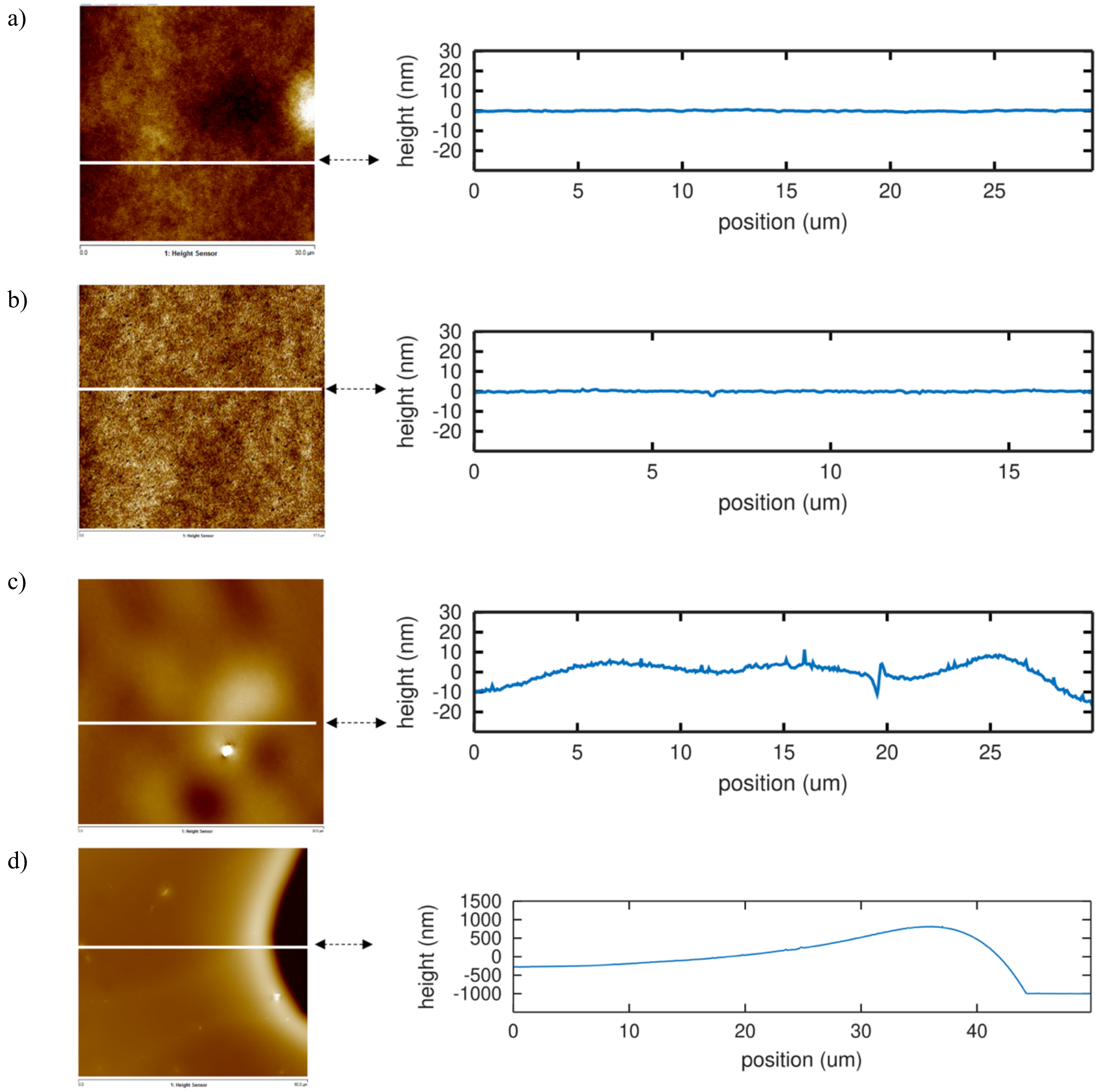

Figure S3. AFM topographes (left) and corresponding height profile along the white line (right) of a silicon surface covered with a layer of PIB (thickness: 690 nm). a) Flat surface after deposition of PIB by spin-coating. b) The surface is still quite flat after immersion in water-acetonitrile (96:4) solution during 1h. c) After immersion in a water-acetonitrile solution of chlorpyrifos during 12 h, the surface of PIB layer is rough, with a distance of peak-to-valley

close to 20 nm. d) Crossing (690 nm) holes are observed after rinsing during 1 h with an acetonitrile-water solution (96:4).

6. AFM images of PECH layer

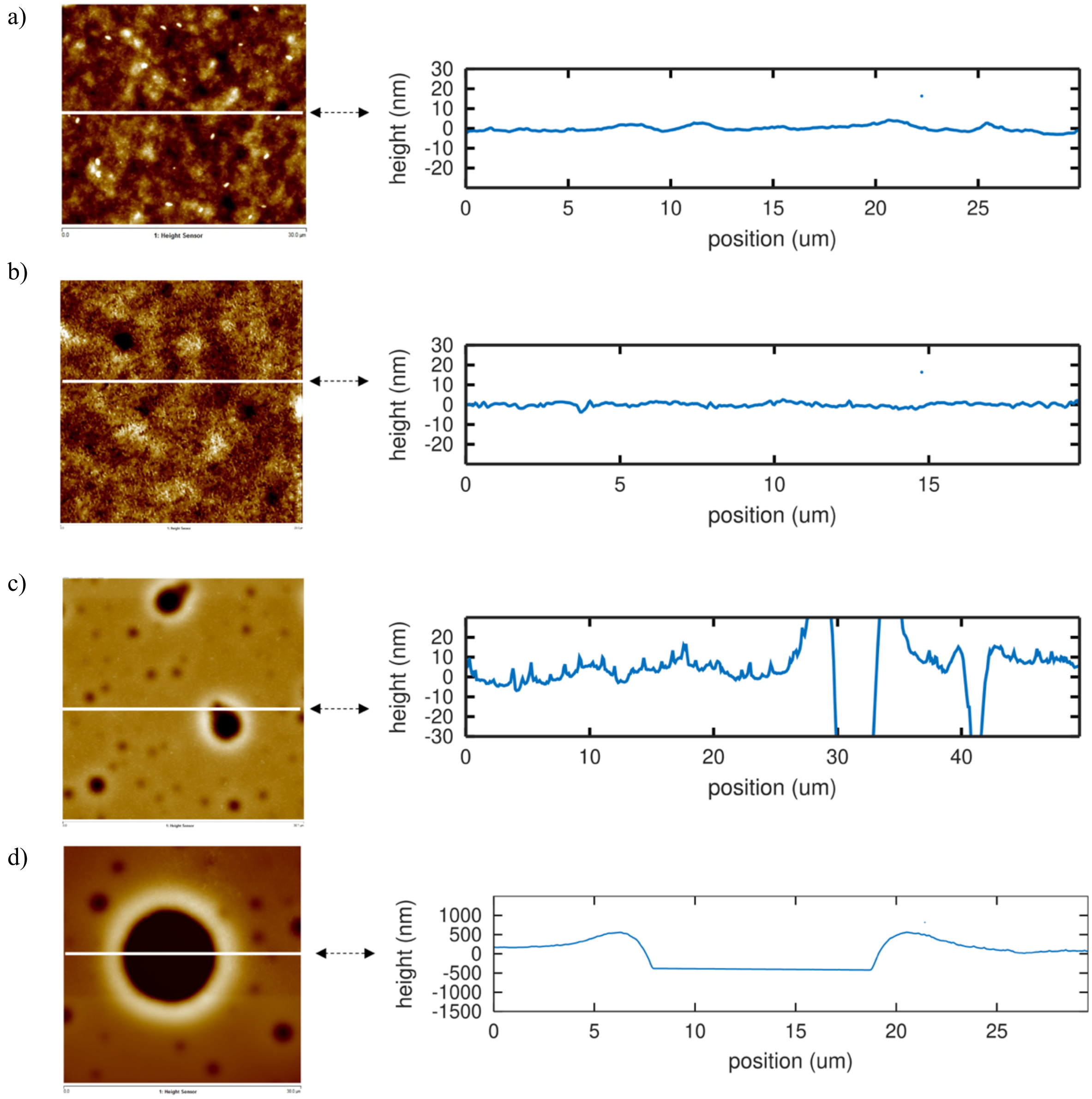

Figure S4. AFM topographes (left) and corresponding height profile along the white line (right) of a silicon surface covered with a layer of PECH (thickness: 480 nm). a) Flat surface after deposition of PECH by spin-coating. b) The surface is still quite flat after immersion in water-acetonitrile (96:4) solution during 1h. c) After immersion in a water-acetonitrile solution of chlorpyrifos during 12 h, the surface of PECH layer is rougher than those observed with PIB or PBMA layers, with deep holes (depth: 200 nm. d) Crossing (480 nm) and deep holes are observed after rinsing during 1 h with an acetonitrile-water solution (96:4).

7. FT-IR spectra of PIB layer

a)

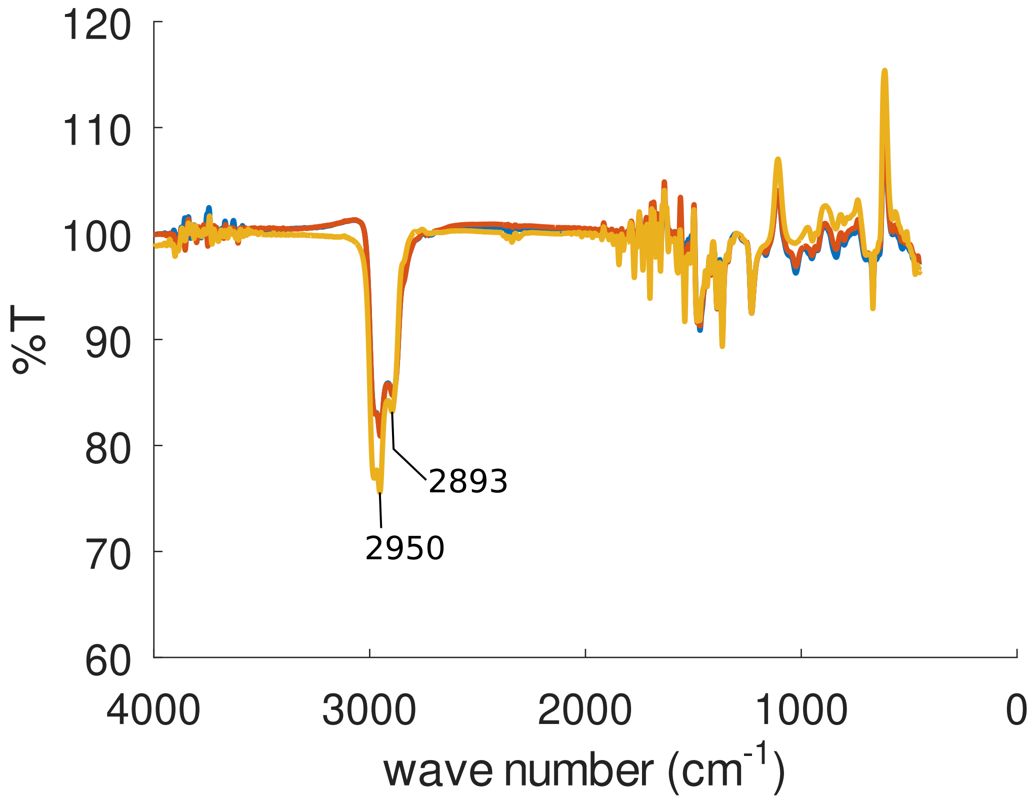

b)

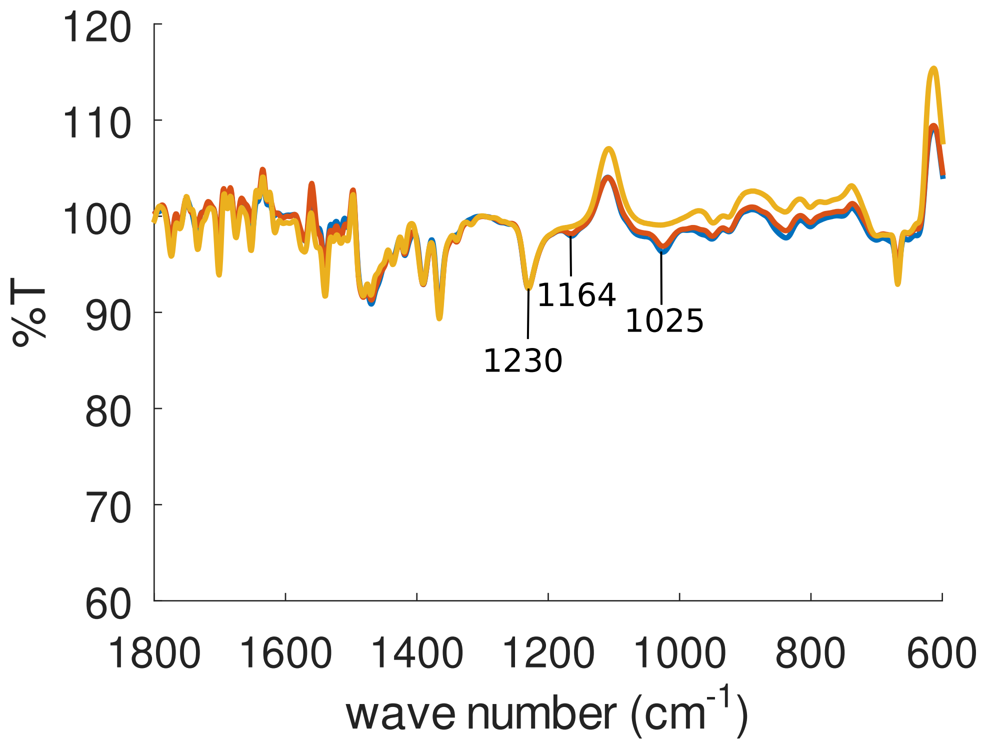

Figure S5. FT-IR spectra of a PIB layer (thickness: 220 nm) deposited on a silicon substrate. In blue, spectrum of PBMA layer after deposition onto silicon substrate, in red, this layer immerged overnight in a water-acetonitrile solution containing 40 mg/L of chlorpyrifos, and in

yellow, this layer washed with water-acetonitrile solution during 1h30. a) full extended spectrum; b) zoom corresponding to 400-1800 cm^{-1} range.

8. FT-IR spectra of PECH layer

a)

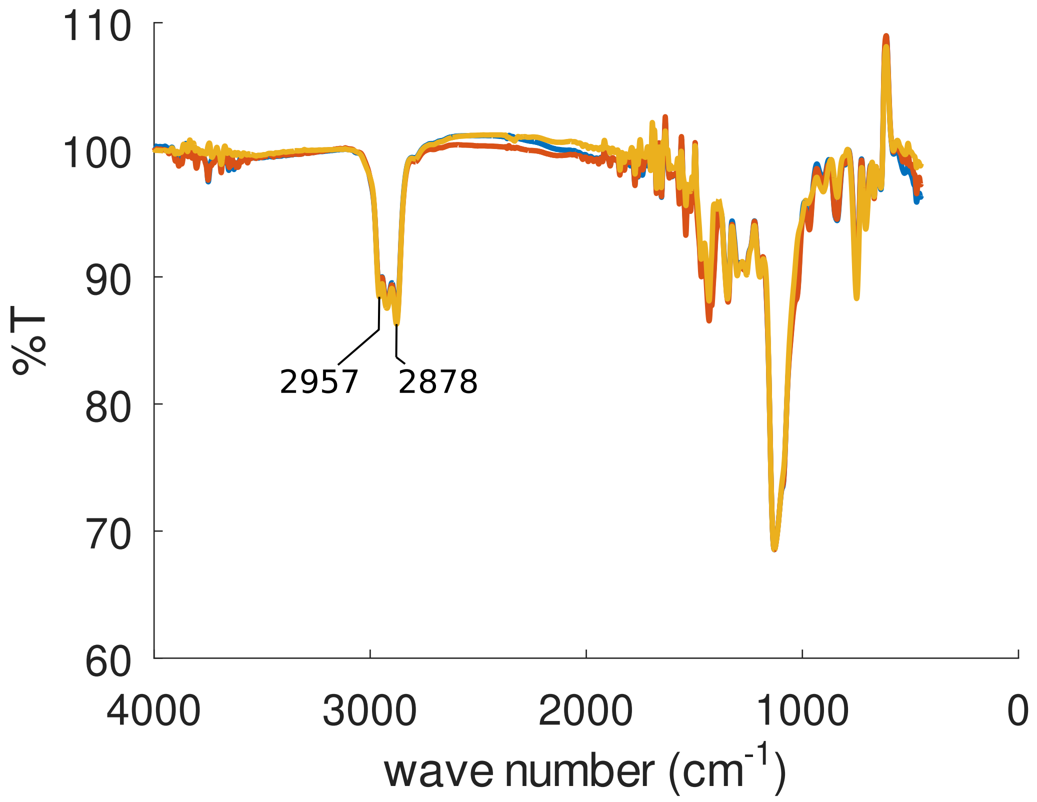

b)

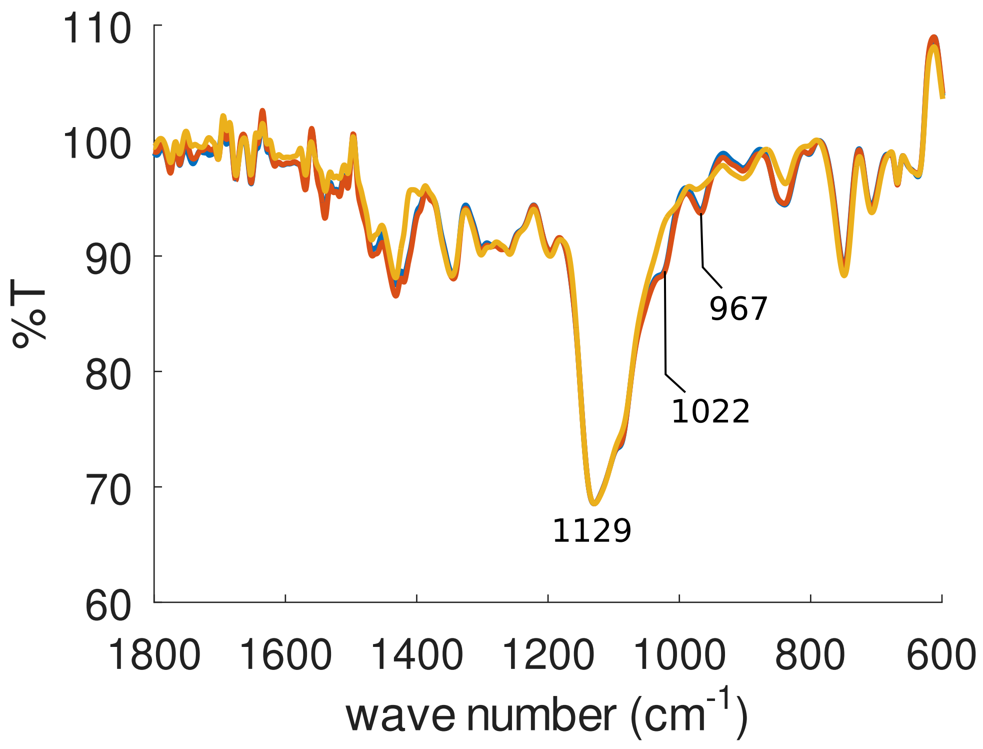

Figure S6. FT-IR spectra of a PECH layer (thickness: 480 nm) deposited on a silicon substrate. In blue, spectrum of PBMA layer after deposition onto silicon substrate, in red, this layer immerged overnight in a water-acetonitrile solution containing 40 mg/L of chlorpyrifos, and in yellow, this layer washed with water-acetonitrile solution during 1h30. a) full extended spectrum; b) zoom corresponding to 400-1800 cm^{-1} range.

a)

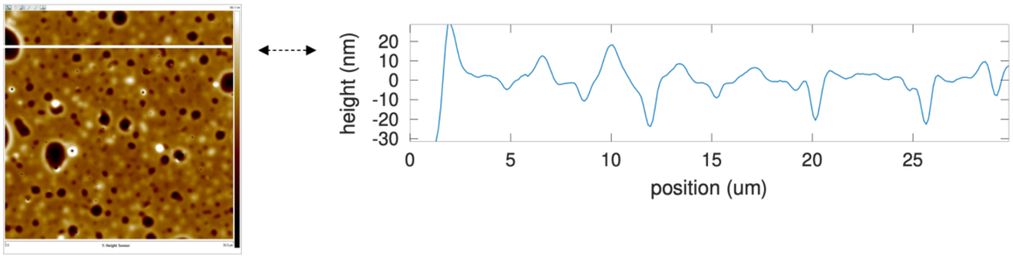

b)

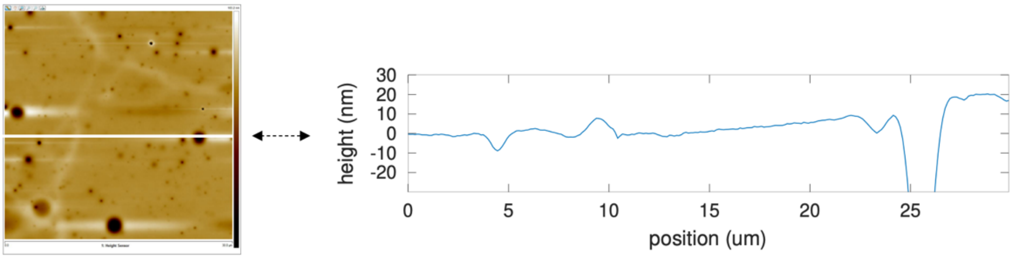

Figure S7. AFM topographies (left) and corresponding height profile along the white line (right) of a silicon surface covered with a spin-coated layer of PBMA (thickness: 500 nm) after immersion a) in water-acetonitrile (96:4) solution and b) in water-acetonitrile (98:2) containing 2 mg of chlorpyrifos during 12h. The presence of acetonitrile strongly increases the surface density of holes. The hole positioned around 25 microns has a deep of 100 nm.
